# Successful treatment of cholecystolithiasis by ERCP: A case report and literature review (with video)

**DOI:** 10.1111/1751-2980.13105

**Published:** 2022-07-22

**Authors:** Ge Zhang, Jie Lin Li, Ming Ji, Shu Tian Zhang, Peng Li, Yong Jun Wang, Yong Dong Wu

**Affiliations:** ^1^ Department of Gastroenterology The Second Affiliated Hospital of Shandong First Medical University Tai'an Shandong Province China; ^2^ Department of Gastroenterology Beijing Friendship Hospital, Capital Medical University Beijing China; ^3^ Beijing Key Laboratory for Precancerous Lesion of Digestive Disease, National Clinical Research Center for Digestive Disease Beijing Digestive Disease Center Beijing China

Dear Editor,

Cholelithiasis is a common disease in clinical practice. Endoscopic retrograde cholangiopancreatography (ERCP) is a standard treatment for common bile duct stones (CBDS). Approximately 10%–18% of patients who undergo cholecystectomy for gallstones have CBDS,^1^ which is generally managed by peri‐ERCP laparoscopic cholecystectomy (LC). Theoretically, ERCP is also technically possible for gallbladder (GB) drainage. However, extraction of GB stones through the tortuous cystic duct is often difficult during ERCP. Here we reported a case of cholecystolithiasis with CBDS that was successfully managed using ERCP. This letter was approved by the Ethical Committee of Beijing Friendship Hospital, Capital Medical University (Beijing, China) (no. 2021‐P2‐423‐01).

A 30‐years‐old man was admitted to the Department of Gastroenterology, Beijing Friendship Hospital on 5 September 2017, complaining of abdominal pain after satiation for 3 days and yellow‐dyed skin and sclera observed the day before the admission. Abdominal computed tomography in another hospital before this admission had shown choledocholithiasis and cholecystolithiasis. Considering significant surgical trauma of LC or laparoscopic common bile duct (CBD) exploration, the patient gave consent to endoscopic treatment. On the same day, ERCP was performed and a small number of sediment stones were removed from the bile duct. The patient was then discharged after symptom relief.

On 29 July 2020 the patient was re‐admitted to the hospital due to abdominal pain after intermittent greasy food consumption during the past two 2 years. The patient was diagnosed with GB stones and chronic cholecystitis. The patient again refused surgery and asked for endoscopic treatment. On 30 July 2020 ERCP was performed, showing multiple GB stones; a plastic stent was placed in the cystic duct under ERCP (Figure 1A). In detail, one end of the stent was located in the GB and the other end at the oral side of the duodenal papilla, through which the obstruction was resolved and the drainage of bile and fine stones from the GB was carried out. The patient was asked to readmit for the removal of the cystic duct stent 3 months after the ERCP.

**FIGURE 1:**
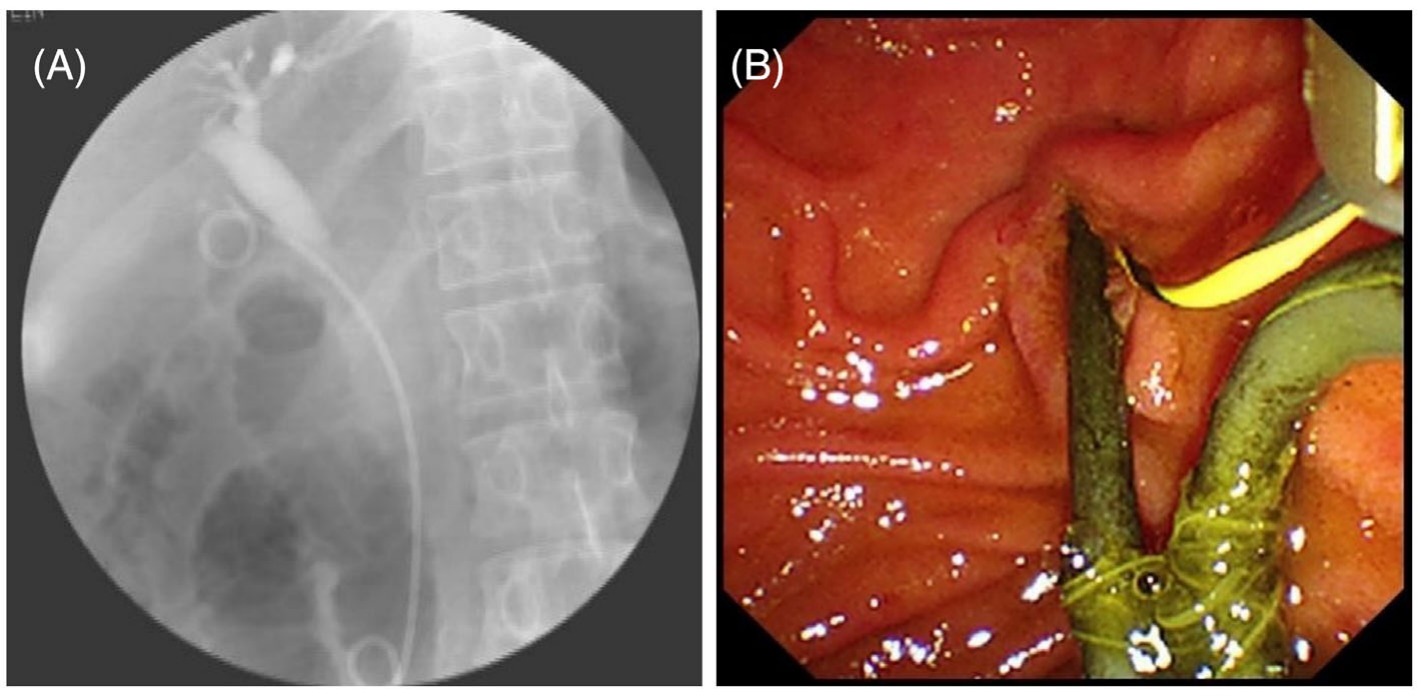
A, Stent was placed in gallbladder under endoscopic retrograde cholangiopancreatography (30 July 2020). B, Plastic stent is visible through duodenoscope (13 April 2021)

Owing to the COVID‐19 pandemic, the patient was admitted 9 months later and ERCP was performed on 13 April 2021. Under duodenoscope, the plastic stent was observed in the descending duodenum (Figure 1B). However, the duodenal incision knife with a guidewire along the stent had difficulty in entering the GB. Therefore, a sliding guidewire thread was used, with radiography showing multiple flocculent stone shadows in the GB. The guidewire was then retained, the double‐pigtail plastic stent was removed, and a 10‐Fr fully covered self‐expandable metal stent (SEMS) of 12 cm in length was placed in the cystic duct. The bile duct was successfully located using the guidewire with an angiography tube to prevent poor intrahepatic bile outflow, and a 7‐Fr double‐pigtail plastic stent (12 cm in length) was placed along the guidewire to the porta hepatis (Figure 2A,B). SpyGlass was directed through the metal stent to enter the GB, and yellowish‐green siltlike stones were observed. In addition, normal saline was used to flush the GB repeatedly, but no massive stones or masses were found. Cholecystography was then performed; no obvious abnormal stone shadow was found in the entire GB. Mild hyperemia of local mucosa of the GB wall was observed, and biopsy forceps was used to obtain tissue sample for histopathology (Video S1), showing that the mucosa of GB was covered with columnar epithelium, with scattered inflammatory cell infiltration and massive lymphocytes; some tissues were squeezed and deformed by the biopsy forceps. After surgery, the patient was treated with antibiotics and fluid infusion and discharged the next day.

**FIGURE 2:**
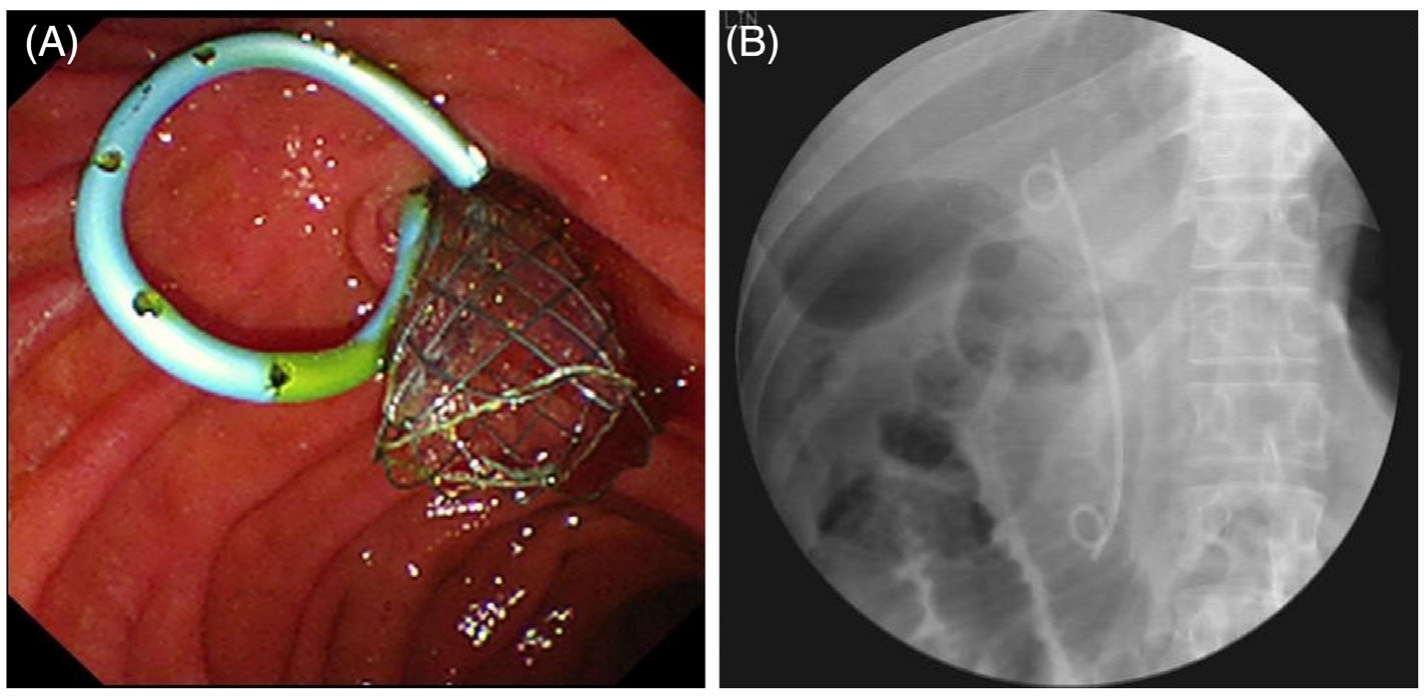
A, Metal stent was placed in the gallbladder and plastic stent in the bile duct (13 April 2021). B, Metal stent in gallbladder and plastic stent in bile duct observed on X‐ray examination (13 April 2021)

On 22 June 2021, the patient was admitted to the hospital to remove the metal stent from the GB. SpyGlass was used to enter the GB through the metal stent (Figure 3A), with multiple flocculent stones found in the GB again (Figure 3B). Repeated saline irrigation showed that the flocculent stones were discharged; larger stones were removed with biopsy forceps (Video S2). The metal stent in the GB duct was removed with a snare device once floccus in the GB was no longer observed. The patient was discharged the next day. A follow‐up phone call was made on 18 April 2022, the patient reported no discomfort such as abdominal distention or abdominal pain.

**FIGURE 3:**
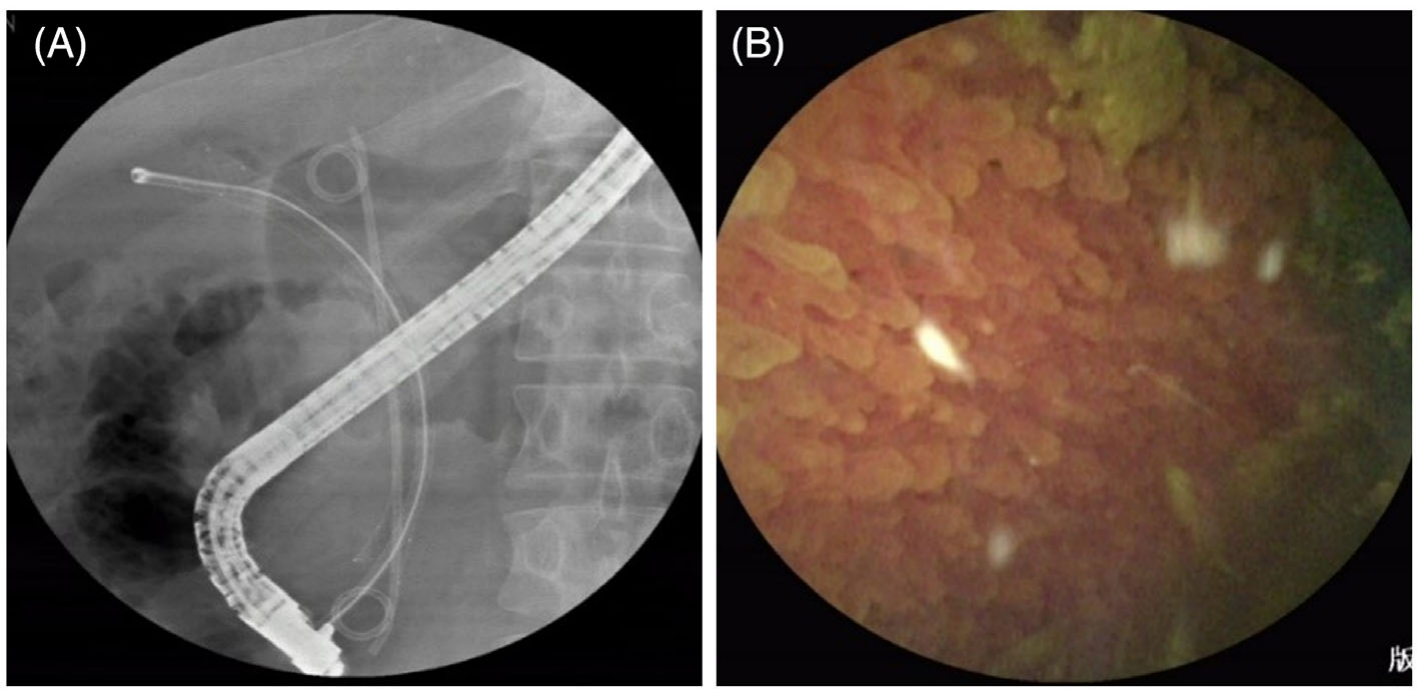
A, X‐ray imaging (22 June 2021) showing successful passage of SpyGlass through metal stent to enter gallbladder. B, Multiple gallbladder flocculent stones observed through SpyGlass (22 June 2021)

Cholecystolithiasis with choledocholithiasis is characterized by a short course of disease and rapidly changing condition, which can easily cause acute cholangitis and cholecystitis that require emergency ERCP or surgical treatment. Thanks to the continuous advances in endoscopic technology in recent years, cholecystolithiasis with choledocholithiasis can be treated with endoscopy and minimally invasive endoscopic technology, achieving satisfactory prognosis.^2^, ^3^

At present, the treatment for cholecystolithiasis with choledocholithiasis has gradually changed from open cholecystectomy plus common bile duct exploration and T‐tube drainage to LC in combination with ERCP or endoscopic sphincterotomy and lithotomy, and LC and laparoscopic CBD exploration. Both treatment strategies can be used to treat cholecystolithiasis complicated with CBDS. Rogers et al^4^ and Ding et al^5^ reported no significant differences between ERCP in combination with LC and LC alone in terms of stone clearance, complication, patient acceptance, and quality of life score. ERCP choledocholithotomy with LC was preferred for patients with choledocholithiasis with cholecystolithiasis as recommended by the Chinese guidelines for ERCP (2018).^6^ For our patients, a less invasive holistic approach was used. First, ERCP was used to remove CBDS, and a plastic cystic duct stent was placed. After the cystic duct was fully expanded, the plastic stent was replaced with a metal stent with a wider inner diameter. Then SpyGlass was used to remove GB stones. Storm et al^7^ reported 51 patients with acute cholecystitis who could not undergo cholecystectomy. ERCP‐guided transpapillary GB drainage with transpapillary GB double‐pigtail stent placement has proven to be a safe and effective long‐term therapy for poor surgical candidates with acute cholecystitis. A study in Japan^8^ showed a successful placement of plastic stents in the GB and bile duct in an elderly patient with cholecystolithiasis complicated by acute cholecystitis. Compared with previous case reports,^7,8^ we not only applied both cystic duct plastic stent and metal stent but also used a cystic duct metal stent and SpyGlass, which guaranteed further stone removal and direct biopsy of the GB wall. After the metal stent was placed in the bile duct, the gallstones could be extracted spontaneously or washed out with normal saline under SpyGlass. We also used biopsy forceps to remove massive flocculent stones, which is suitable for relatively small calculi. For large calculi, we speculated that it was possible to use a choledochoscope for laser/electrohydraulic lithotripsy via the cystic duct metal stent or basket extraction for small calculi with a fully covered SEMS.

At present, LC is the standard treatment for gallstones. Although the complication rate of LC is relatively low, bile duct injury, bile fistula, gastrointestinal injury, choledocholithiasis, and abdominal infection can still occur.^8^ Functional complications, such as postcholecystectomy syndrome,^9^, ^10^ can also occur after LC, causing epigastric pain, diarrhea, dyspepsia, constipation, etc. However, using endoscopic lithotomy, not only can the GB be preserved but organic injury can also be reduced. In addition, the GB wall can be observed directly through SpyGlass. For GB polyps, adenomas and cancers, not only can the nature of lesions be determined under direct endoscopic view, but the “target” can also be accurately biopsied under direct vision, thereby providing pathological evidence for the identification of benign and malignant GB diseases. The most significant difficulty with ERCP is the physiological and anatomical characteristics of the cystic duct, whose diameter is spiral and twisted. Therefore, making the guidewire enter the GB through the tortuous and small cystic duct has always been difficult for clinicians.

The success achieved in our patient makes it possible to promote endoscopic diagnosis and treatment of GB diseases in clinical practice. This case paves the way for the endoscopic resection of gallstones, benign GB adenoma, and even early cancer. However, ERCP is related to complications such as postoperative pancreatitis, bleeding, perforation, and infection, in which postoperative pancreatitis is the most common complication with a reported incidence of up to 9.7%, and even 14.7% for high‐risk groups.^11^ However, as an endoscopic technique, ERCP maintains the physiological and anatomical structure of the patient, thereby avoiding surgical complications and reducing the risk of infection.

Endoscopic ultrasound‐guided gallbladder drainage (EUS‐GBD) can also be considered for symptomatic patients with cholelithiasis or acute cholecystitis who still encounter unresolved bile duct obstruction after ERCP, although adverse reactions have been noted following EUS‐GBD. In a systematic review of 189 patients with acute cholecystitis treated with EUS‐GBD, the recurrence rate of postoperative cholecystitis was 5.1%, the incidence of gastrointestinal bleeding and stent displacement was 2.6% and 1.1%, respectively.^12^ A study^13^ reported a preliminary discussion on the removal of biliary lithotomy through gastric endoscopy in treating GB calculi. Endoscopic transgastric GB–preserving cholecystolithotomy proved to be an effective and safe way to treat cystic stones that can preserve GB function and is minimally invasive. However, dealing with calculi and avoiding residues and recurrence require further exploration. In addition, long‐term curative effects require further observation. ERCP stone removal using a cystic duct metal stent, endoscopic stone removal via gastric GB preservation, and EUS‐GBD are all minimally invasive. Large trials with long‐term postoperative follow‐up are warranted in the future.

ERCP may replace LC and other treatment strategies, both surgical or non‐surgical, for cholecystolithiasis with choledocholithiasis in the future. In addition, endoscopic GB wall biopsy not only provides pathological support for the differentiation of benign and malignant diseases of the GB but also offers the possibility of endoscopic resection of GB lesions.

## Supporting information


**Video S1** Observation of gallbladder mucosa by using SpyGlass and tissue sample was obtained for histopathological examination.

**Video S2** Stones were removed by repeated saline irrigation.
